# Investigation of the Key Pharmacological Activities of* Ficus racemosa* and Analysis of Its Major Bioactive Polyphenols by HPLC-DAD

**DOI:** 10.1155/2016/3874516

**Published:** 2016-12-26

**Authors:** Salma Akter Sumi, Md. Afjalus Siraj, Amir Hossain, Md. Sagir Mia, Seagufta Afrin, Md. Mustafizur Rahman

**Affiliations:** ^1^Pharmacy Discipline, Life Science School, Khulna University, Khulna 9208, Bangladesh; ^2^Department of Pharmaceutical Sciences, Daniel K. Inouye College of Pharmacy, University of Hawaii at Hilo, Hilo, HI 96720, USA; ^3^Pharmacy Department, Jahangirnagar University, Dhaka 1342, Bangladesh

## Abstract

*Objective*. Oxidative stress leads to numerous physiological disorders including infectious diseases, inflammation, and cancer. The present study was carried out to investigate antioxidant, antibacterial, and cytotoxic activity of methanol crude extract of leaves and fruits of the* Ficus racemosa* (LCME and FCME, resp.) and to analyse its major bioactive polyphenols by HPLC-DAD.* Methods*. Antioxidant capacity of the extracts was evaluated by DPPH free radical scavenging, reducing power, total phenolic, total flavonoid, total tannin content assay, superoxide radical, hydroxyl radical, and hydrogen peroxide scavenging assay. Identification and quantification of bioactive polyphenols were done by HPLC-DAD method. Antibacterial activity was tested by “disc diffusion” method. Brine shrimp lethality assay was carried out to check the cytotoxic potential.* Result*. Both LCME and FCME showed DPPH scavenging ability and concentration dependent reducing power activity. They had phenolic content, flavonoid content, and tannin content. Both the extracts showed superoxide radical scavenging ability, hydroxyl radical scavenging ability, and hydrogen peroxide scavenging ability. HPLC analysis of LCME and FCME indicated the presence of significant amount of gallic acid along with other phenolic constituents.* Conclusion*. Significant amount of gallic acid along with other phenolic constituents might have played an important role in the observed antioxidant, antibacterial, and cytotoxic activity.

## 1. Introduction

Many plant species have been identified to have potent pharmacological activity against diseases [1, 2]. According to WHO (World Health Organization), 80% of the world's population from developing countries still relies on plant derived medicines for the treatment and approximately 25% of modern drugs used in USA are plant derivatives [3]. Few members of genus* Ficus* have significant medicinal values.* Ficus racemosa* Linn. (family: Moraceae) is a well-known medicinal plant which is native to South-East Asia. This plant is called Udumbara in Bangladesh [4, 5]. Different parts like root, bark, and stem have been used to isolate various chemical constituents. The leaf of* F. racemosa* contains tetra triterpene, glauanol acetate, and racemosic acid. The fruit have glauanol, hentriacontane, *β* sitosterol, tiglic acid, *β*-sitosterol, cycloartenol, cycloeuphordenol, euphol, euphorbinol, isoeuphorbol, palmitic acid, and so forth [6].* F. racemosa* has various uses in traditional medicine in the subcontinent for treating an array of diseases. The bark has good hypolipidemic, antidiuretic, anthelmintic, anticholinesterase, memory enhancing, and analgesic activities. It was also found to be useful in diabetes, dysentery, piles, and urological disorders. The root is beneficial in treatment of diabetes, dysentery, and various inflammatory glandular enlargements. The latex is good for haemorroids, traumatic swelling, and vaginal disorders. The leaf showed antihyperglycemic, anti-inflammatory, antibacterial and hepatoprotective effects. The combination of bark and leaf infusion is a good form of mouth wash. Fruits are astringents and can be used in kidney and spleen disease [7, 8].

Despite having a number of pharmacological studies, many scientists are still interested about the leaf and fruit parts of* F. racemosa* as both of them are very rich in bioactive chemical compounds. The aim of the present study was to investigate and evaluate the antioxidant, antibacterial, and cytotoxic activities of the fruit and leaf part of* F. racemosa* and analysis of its major bioactive polyphenols by HPLC.

## 2. Methods

### 2.1. Plant Collection and Extraction

In the present study,* F. racemosa* was collected from the Khulna district, Bangladesh, and identified by the experts at Bangladesh National Herbarium, Dhaka, Bangladesh. A voucher specimen (DACB 38388) has been submitted there for future reference. After collection, leaf and fruit parts were grinded into a coarse powder form by grinder. The plant part was then extracted by hot extraction with the help of Soxhlet apparatus. 250 g of leaf and fruit powder was extracted with methanol.

### 2.2. Chemicals

Arbutin (AR), gallic acid (GA), hydroquinone (HQ), (+)-catechin hydrate (CH), vanillic acid (VA), caffeic acid (CA), syringic acid (SA), (−)-epicatechin (EC), vanillin (VL),* p*-coumaric acid (PCA),* trans*-ferulic acid (FA), myricetin (MC), ellagic acid (EA),* trans*-cinnamic acid (TCA), rosmarinic acid (RA), benzoic acid (BA), quercetin (QU), rutin hydrate (RH), and kaempferol (KF) were purchased from Sigma-Aldrich (St. Louis, Missouri, United States). Acetonitrile (HPLC), methanol (HPLC), acetic acid (HPLC), ethanol, Folin-Ciocalteu's reagent, ascorbic acid, trichloroacetic acid, potassium ferricyanide, sodium carbonate, ferric chloride, DMSO (dimethyl sulfoxide), and acetic acid were obtained from Merck (Darmstadt, Germany). Diclofenac sodium and vincristine sulphate were obtained from Beximco Pharmaceuticals Ltd. and Cipla Pharmaceuticals India, respectively.

### 2.3. Antioxidant Activity Test

#### 2.3.1. DPPH Free Radical Scavenging Activity

Stock solution of LCME and FCME was prepared and serially diluted to obtain different concentrations. As standard antioxidant, ascorbic acid was used. 1 mL of each concentration was added to 0.004% DPPH of 2 mL solution. The mixture was kept in dark (25°C) for 30 minutes to complete the reaction. The absorbance was measured at 517 nm in a double beam UV visible spectrophotometer. IC_50_ (50% inhibitory concentration) value was determined from percent inhibition versus concentration graph [9].

#### 2.3.2. Reducing Power Assay

To determine the reducing power of LCME and FCME, different concentrations (12.5–800 *μ*g/mL) of extracts and standard (ascorbic acid) were mixed with 2.5 mL, 1% potassium ferricyanide (K_3_Fe(CN)_6_), and 2.5 mL, 0.2 M phosphate buffer (pH 6.6) with a gentle shaking. At 50°C temperature, mixtures were then incubated for about 20 mins. After the incubation, the mixtures were kept at room temperature. Then, 10% trichloroacetic acid of 2.5 mL was added followed by centrifugation at 3000 rpm for 10 min. 0.1% ferric chloride (0.5 mL) and distilled water (2.5 mL) were mixed with the supernatant of 2.5 mL. Absorbance was then measured at 700 nm after 5 min [10].

#### 2.3.3. Determination of Total Phenolic Content

The plant extract (0.5 g) was mixed with 80% aqueous methanol (50 mL) and sonicated for about 20 min. A part of 2 mL was taken from it and centrifuged for 15 min at 14,000 rpm. Folin-Ciocalteu's reagent is used for the determination of total phenolic content. Standard gallic acid solutions were prepared by serial dilution (20–100 *μ*g/mL). 1 mL of each concentration of gallic acid solutions and plant extracts was added with 9 mL distilled water. 1 mL of Folin-Ciocalteu's reagent was added to each concentration with continuous shaking. After 5 min, 10 mL of 7% Na_2_CO_3_ was added and adjusted with distilled water to make the final volume of 25 mL. It was kept for 30 min to complete reaction. At the end, an absorbance was estimated at 750 nm [11].

#### 2.3.4. Determination of Total Flavonoid Content

Three hundred microliter (0.3 mL) of each plant extract and standard (Rutin) were mixed with 30% methanol (3.4 mL), 0.5 M NaNO_2_ (0.15 mL), and 0.3 M AlCl_3_·6H_2_O (0.15 mL). After 5 min, 1 mL 1 M NaOH was added. Then, the solution was mixed properly and the absorbance was measured at 506 nm. The total flavonoids were expressed as mg equivalents per gram of dried fraction [12].

#### 2.3.5. Determination of Total Tannin Content

The total tannin content was measured by Folin-Ciocalteu's method. For doing this, 0.5 mL of Folin Phenol reagent and 7.5 mL of distilled water were mixed with 0.1 mL sample extract and then mixed with 1 mL of 35% Na_2_CO_3_ solution and diluted to 10 mL with distilled water. Then, the mixture was shaken properly and kept at room temperature for 30 min. The reference standard solution of gallic acid (20–100 *μ*g/mL) was prepared using the same procedure. Absorbance of the sample and standard solutions were measured against the blank at 725 nm with an UV-visible spectrophotometer. The tannin content was expressed in terms of mg of GAE/g of extract [13, 14].

#### 2.3.6. Superoxide Radical Scavenging Assay

The reaction mixture contained 1 mL of NBT solution (312 *μ*M) prepared in phosphate buffer of pH 7.4 and 1 mL of NADH solution (936 *μ*M) prepared in phosphate buffer of pH 7.4. 0.1 mL of plant extracts and standards diluted in different concentrations were added. Finally, reaction was amplified by adding 100 *μ*L PMS solution (120 *μ*M prepared in phosphate buffer adjusting pH 7.4) to the mixture. The reaction mixture was incubated at 25°C for 5 min and absorbance was taken at 560 nm [15].

#### 2.3.7. Hydroxyl Radical Scavenging Assay

Hydroxyl radical scavenging activity was evaluated on the basis of scavenging of hydroxyl free radical produced in the Fenton reaction by standard (Quercetin), LCME, and FCME. The reaction combination in ultimate volume of 1.0 mL contains 200 *μ*L of 1.04 mM EDTA and 200 *μ*M FeCl_3_ (1 : 1 v/v), 100 *μ*L of 2-deoxy 2-ribose, 100 *μ*L of 1.0 mM hydrogen peroxide (H_2_O_2_), 100 *μ*L of 1.0 mM ascorbic acid, and 500 *μ*L of the fractions at various concentrations ranging from 50 to 800 *μ*g/mL in buffer. The samples tested were kept at 37°C for 1 hour. On the substrate, deoxyribose was estimated by the thiobarbituric acid test as the free radical damage executed on it. Then, incubation was done at 1000°C for 20 min on the mixture of 1.0 mL trichloroacetic acid (2.8%) and 1 mL of thiobarbituric acid (1%). After that, an absorbance was determined at 532 nm [16].

#### 2.3.8. Hydrogen Peroxide Scavenging Assay

For the completion of hydrogen peroxide scavenging assay, a hydrogen peroxide solution of 2 mM/L was arranged with standard phosphate buffer having pH 7.4. the prepared hydrogen peroxide solution of 0.6 mL was mixed with the diverse concentration of fractions ranging from 25 to 400 *μ*g/mL in distilled water. Absorbance was determined at 230 nm after 10 min against a blank solution containing phosphate buffer without hydrogen peroxide. The scavenging activity counting its percentage at different concentrations of LCME and FCME was estimated and a standard (*α*-tocopherol) is used for the comparison of the measured IC_50_ values [17].

### 2.4. HPLC Detection and Quantification of Polyphenolic Compounds

It is done in the methanol extract by HPLC-DAD analysis. It was carried out on a Dionex UltiMate 3000 system equipped with quaternary rapid separation pump (LPG-3400RS) and photodiode array detector (DAD-3000RS). Separation was performed using Acclaim® C_18_ (5 *μ*m) Dionex column (4.6 × 250 mm) at 30°C with a flow rate of 1 mL/min and 20 *μ*L injection volume. The mobile phase contained three solvents' system involving acetonitrile (solvent A), acetic acid solution pH 3.0 (solvent B), and methanol (solvent C) with the gradient elution program of 5%A/95%B (0–5 min), 10%A/90%B (6–9 min), 15%A/75%B/10%C (11–15 min), 20%A/65%B/15%C (16–19 min), 30%A/50%B/20%C (20–29 min), 40%A/30%B/30%C (30–35 min), and 100%A (36–40 min). The UV detector was set to 280 nm for 22.0 min, changed to 320 nm for 28.0 min, again changed to 280 nm for 35 min and finally to 380 nm for 36 min, and held for the rest of the analysis period while the diode array detector was set at an acquisition range from 200 nm to 700 nm. For the preparation of calibration curve, a standard stock solution was prepared in methanol containing AR and ECA (5 *μ*g/mL each), GA, HQ, VA, RA, and MC (4 *μ*g/mL each), CA, SA, VL, and FA (3 *μ*g/mL each), PCA, QU, and KF (2 *μ*g/mL each), CH and EA (10 *μ*g/mL each), TCA (1 *μ*g/mL), RH (6 *μ*g/mL), and BA (8 *μ*g/mL). The extract was dissolved in methanol making a concentration of 10 mg/mL. Before starting HPLC analysis, all the solutions prepared were filtered through a 0.20 *μ*m syringe filter and then degassed in an ultrasonic bath (Hwashin, Korea) for 15 min. All the calculations including peak integration, data acquisition, and calibrations were done with Dionex Chromeleon software (version 6.80 RS 10) [18, 19].

### 2.5. Antibacterial Activity Test

The antibacterial activity was investigated by disc diffusion assay. The microorganisms from the stock were transferred onto nutrient agar plates and the inoculated overnight at 37°C. Using a sterile loop, small portion of the subculture was transferred into test tube containing nutrient broth and incubated 4 h at 37°C until the growth reached log phase. Nutrient agar media were then set for the standard inoculum suspension and allowed for solidification. Discs (BBL, Cockeysville, USA) impregnated with plant extract (250 and 500 *μ*g/disc), standard antibiotic disc (Tetracycline 30 *μ*g/disc, Oxoid Ltd., UK), and blank (methanol) were placed on the Petri dishes with sterile forceps and gently pressed to ensure contact with the inoculated agar surface. At last, the inoculated plates were incubated at 37°C for 18 h and the zone of inhibition was determined in millimeters [20].

### 2.6. Cytotoxic Activity Test

For the cytotoxic activity test,* Artemia salina* (Brine shrimp) was taken. Around one spoon of cyst of brine shrimp was hatched for about 48 hours in saline water. The saline water was prepared by dissolving 30 mg pure NaCl and 53 mg table salt into 1.5 L water solution of different concentrations that was prepared with the extract by using dimethyl sulfoxide (DMSO) as solvent. A set of eight test tubes were used where 10 shrimps were taken and a solution of different concentration was applied on it. At last, the final volume was adjusted with saline water and kept for 24 hours. Chloramphenicol was used as standard at a concentration of 200 *μ*g/mL. For the preparation of the result, percentage of mortality of the brine shrimp nauplii was estimated to determine LC_50_ (lethal concentration) [20].

### 2.7. Statistical Analysis

Data were presented as mean ± standard deviation (SD). One-way ANOVA followed by Dunnett's test was performed and the results were considered statistically significant when *p* < 0.05.

## 3. Results

### 3.1. Antioxidant Activity

#### 3.1.1. DPPH Radical Scavenging Activity

**Figure 1:**
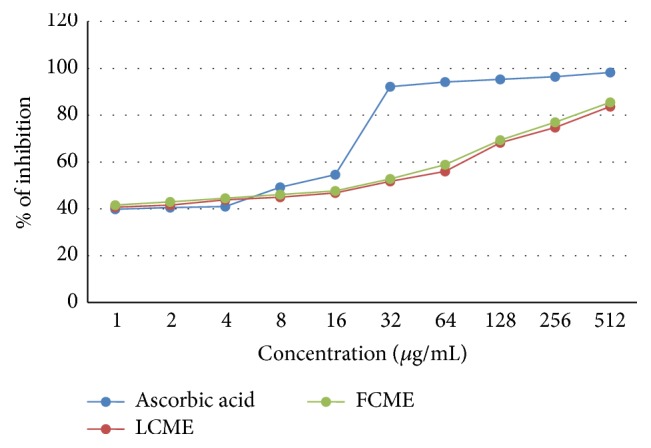
DPPH free radical scavenging activity of methanol crude extract of the leaf and fruit parts of* F. racemose*.

In the DPPH free radial scavenging assay, the leaf part of methanol crude extract (LCME) exhibited IC_50_ = 10.29 *μ*g/mL whereas the fruit part of methanol crude extract (FCME) exhibited IC_50_ = 8.59 *μ*g/mL which were comparable to the standard ascorbic acid (IC_50_ = 4.15 *μ*g/mL) (Figure 1).

#### 3.1.2. Reducing Power

**Figure 2:**
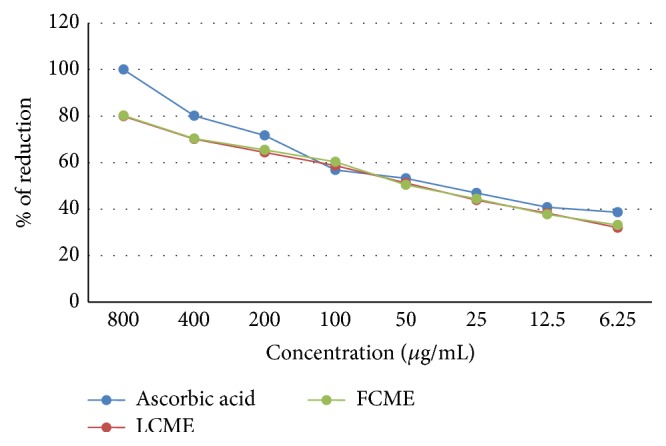
Reducing power of methanol crude extract of the leaf and fruit parts of* F. racemose*.

In the reducing power assay, the LCME displayed RC_50_ = 45.564 *μ*g/mL and FCME displayed RC_50_ = 40.443 *μ*g/mL, which were comparable to the standard ascorbic acid (RC_50_ = 27.589 *μ*g/mL) (Figure 2).

#### 3.1.3. Total Phenolic Content

The total phenolic content of LCME and FCME was 20.2 and 26.2 mg GAE/g of dry extract, respectively (Table 1).

#### 3.1.4. Total Flavonoid Content

The total flavonoid content for LCME and FCME was 22.81 and 10.63 mg QE/g of dry extract, respectively (Table 1).

#### 3.1.5. Total Tannin Content

The total tannin content for LCME and FCME was 19.72 and 21.39 mg sGAE/g of dry extract, respectively (Table 1).

#### 3.1.6. Superoxide Radical Scavenging Activity

**Figure 3:**
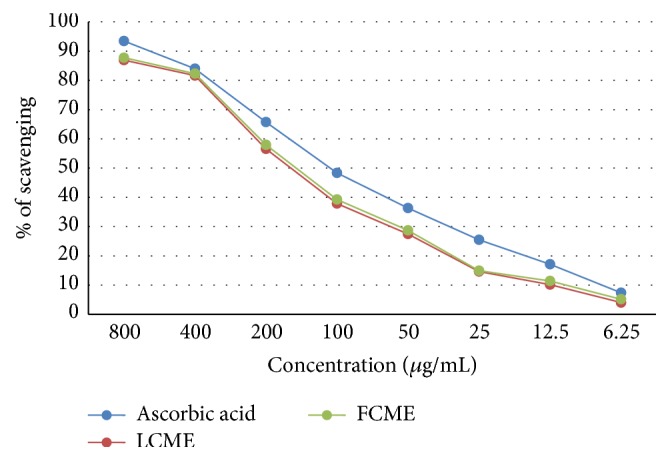
Superoxide radical scavenging activity of methanol crude extract of the leaf and fruit parts of* F. racemosa*.

In this assay, the LCME displayed SC_50_ = 130.104 *μ*g/mL and the FCME displayed SC_50_ = 122.264 *μ*g/mL, which were comparable to standard ascorbic acid (SC_50_ = 83.003 *μ*g/mL) (Figure 3).

#### 3.1.7. Hydroxyl Radical Scavenging Activity

**Figure 4:**
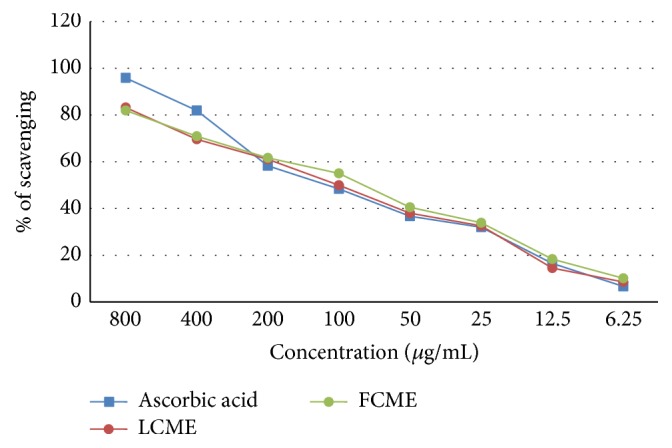
Hydroxyl radical scavenging activity of methanol crude extract of the leaf and fruit parts of* F. racemosa*.

In this assay, the LCME displayed SC_50_ = 103.163 *μ*g/mL and FCME displayed SC_50_ = 91.353 *μ*g/mL, which were comparable to standard ascorbic acid (SC_50_ = 84.148 *μ*g/mL) (Figure 4).

#### 3.1.8. Hydrogen Peroxide Scavenging Activity

**Figure 5:**
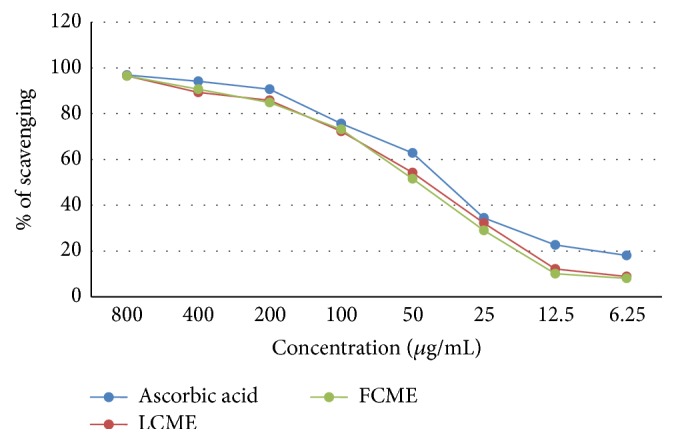
Hydrogen peroxide scavenging activity of methanol crude extract of the leaf and fruit parts of* F. racemosa*.

In this assay, the LCME exhibited SC_50_ = 48.80 *μ*g/mL and the FCME exhibited SC_50_ = 51.825 *μ*g/mL which were comparable to standard ascorbic acid (SC_50_ = 36.058 *μ*g/mL) (Figure 5).

### 3.2. Quantification of Polyphenolic Compounds by HPLC

**Figure 6:**
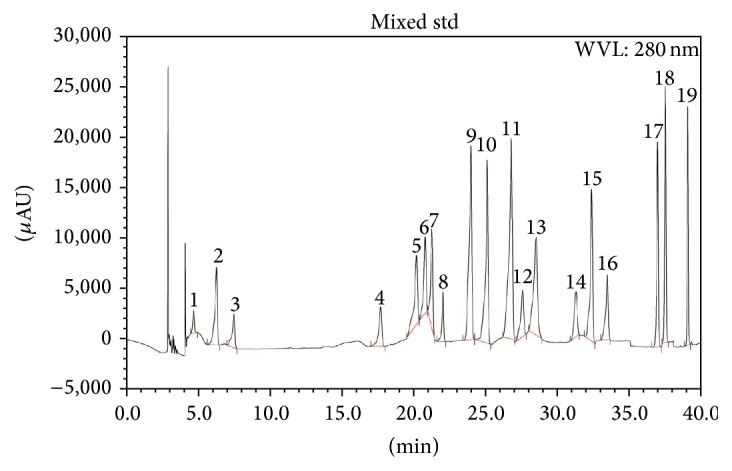
HPLC chromatogram of a standard mixture of polyphenolic compounds. Peaks: 1, arbutin; 2, gallic acid; 3, hydroquinone; 4, (+)-catechin; 5, vanillic acid; 6, caffeic acid; 7, syringic acid; 8, (−)-epicatechin; 9, vanillin; 10,* p*-coumaric acid; 11,* trans*-ferulic acid; 12, rutin hydrate; 13, ellagic acid; 14, benzoic acid; 15, rosmarinic acid; 16, myricetin; 17, quercetin; 18,* trans*-cinnamic acid; 19, kaempferol.

**Figure 7:**
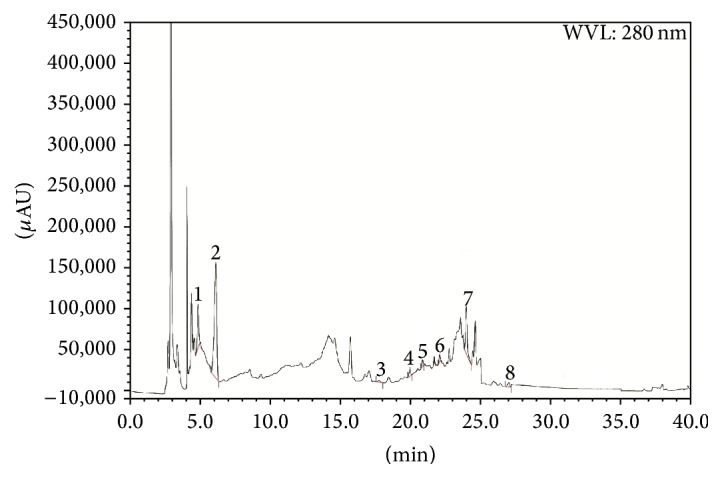
HPLC chromatogram of the methanolic crude extract of* Ficus racemosa* leaf (LCME). Peaks: 1, (AR); 2, gallic acid (GA); 3, (+)- (CH); 4, vanillic acid (VA); 5, caffeic acid (CA); 6, (–)- (ECA); 7, vanillin (VL); 8,* trans*-ferulic acid (FA).

**Figure 8:**
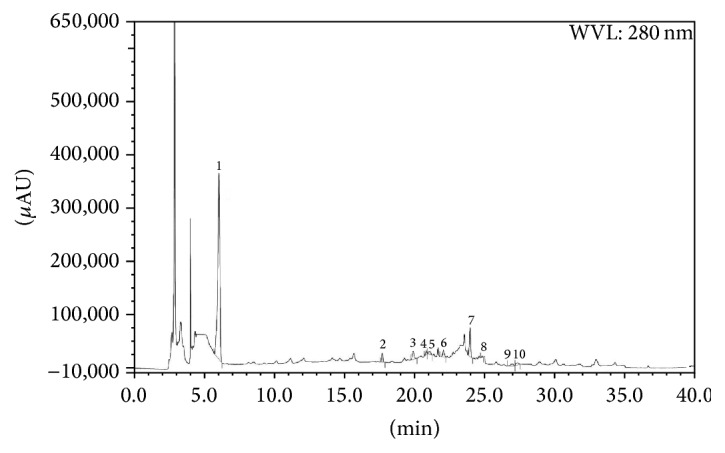
HPLC chromatogram of methanolic crude extract of* Ficus racemosa* fruit (FCME). Peaks: 1, gallic acid (GA); 2, (+)-catechin hydrate (CH); 3, vanillic acid (VA); 4, caffeic acid (CA); 5, syringic acid (SA); 6, (–)-epicatechin (ECA); 7, vanillin (VL); 8,* p*-coumaric acid (PCA); 9,* trans*-ferulic acid (FA); 10, rutin hydrate (RH).

HPLC analysis indicated that both LCME and FCME have significant amount of gallic acid content (37.82 and 50.11 mg/100 g of dry extract). LCME also contained trace amount of arbutin and epicatechin (23.91 and 20.75 mg/100 g of dry extract, resp.) whereas FCME contained trace amount of catechin hydrate and epicatechin (25.34 and 22.14 mg/100 g of dry extract, resp.) (Tables 4 and 5, Figures 6, 7, and 8).

### 3.3. Antibacterial Activity

The leaf and fruit part of methanol crude extract showed moderate antibacterial activity mostly against tested Gram negative (−) bacteria (Tables 2 and 3).

### 3.4. Cytotoxic Activity

**Figure 9:**
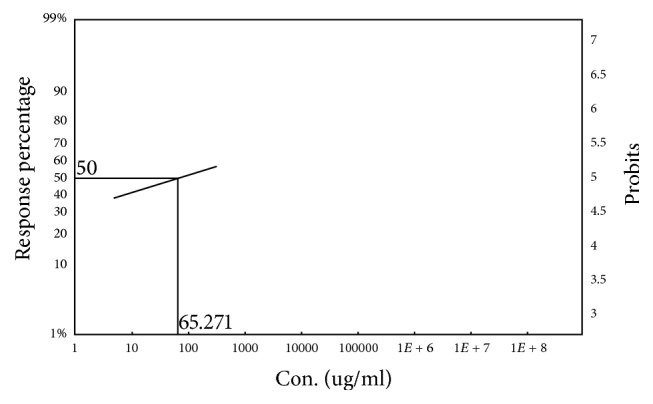
LC_50_ value of methanol crude extract of the leaf of* F. racemosa* (LCME) by using Ldp Line software.

**Figure 10:**
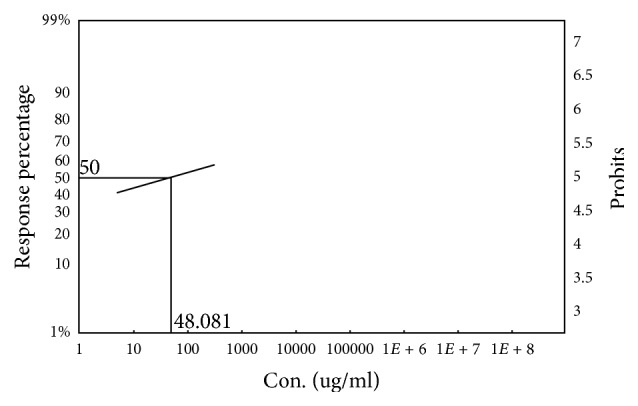
LC_50_ value of methanol crude extract of the fruit of* F. racemosa* (FCME) by using Ldp Line software.

In the brine shrimp lethality bioassay, the 50% lethal concentration of the both leaf and fruit part of* Ficus racemosa* methanol crude extract was 65.271 *μ*g/mL and 48.081 *μ*g/mL, respectively, which was significant and comparable to standard vincristine sulphate (LC_50_ = 0.229 *μ*g/mL) (Figures 9 and 10).

## 4. Discussion

We were interested to run the series of in vitro antioxidant activity test of methanol crude extract of* F. racemosa* leaf and fruit after initially having two positive results. Both of the extracts were found to have tannin in qualitative phytochemical assay. Later on, we also found a significant amount of tannin from both of the extracts. Previous report has shown that tannins have antioxidant activity [21]. Moreover, we carried out qualitative thin layer chromatography (TLC) assay using different solvent systems. Both the extracts showed the free radical scavenging properties indicated by the presence of moderate yellow spot on a purple background on the TLC plate. Generally, antioxidants of the plant parts contain phenolic moiety. For the reasons of the resonance constancy of the phenoxy free radical, phenolic compounds are able to give electrons to the reactive one and create a chain reaction [22].

In the DPPH scavenging assay, antioxidant compounds cause reduction of alcoholic DPPH solution due to their hydrogen-donating capacity [23–25]. Therefore, DPPH radical scavenging activity of extract might be attributed to a presence of some compounds having direct role in trapping free radicals by donating hydrogen atoms. Reducing power capacity may provide a key indication about the antioxidant capacity of a compound [26]. The presence of reductone is related with the reducing power capacity of extracts. The extract also contained a significant amount of phenolic and flavonoid. The presence of hydroxyl group indicates free radical scavenging activity of polyphenolic compounds. Flavonoids are also useful to prevent and treat cardiovascular disease, neurodegenerative problems, and cancer. Their planar structure, number, and position of their hydroxyl groups as well as the presence of the C2-C3 double bond are essential for metal chelation, antioxidant, and free radical scavenging activities [27]. Hydroxyl radical is a potent cytotoxic agent and considered as the most reactive radical produced in living system and responsible for enormous biological damage [28]. Hydrogen peroxide involved in the inactivation of different enzymes by oxidation of essential thiol groups can initiate the generation of hydroxyl radical [29, 30]. The methanol extract was able to neutralize H_2_O_2_ in a concentration-dependent manner, which could be seen by its graded increase in percentage inhibition.

Antibacterial assessment demonstrates whether any species under investigation possess inhibitory activity against bacterial species. In this experiment, the methanol crude extracts were found to have prominent inhibitory property against several pathogenic bacterial species compared to the standard drug kanamycin. Additionally, the results also exhibited that the plant extract possesses a potent cytotoxic activity.

We performed HPLC analysis to justify the correlation between the chemical compounds and the pharmacological activities of this plant. Among nineteen tested polyphenolic compounds, both of the extracts had a significant amount of gallic acid. Having the H-atoms in phenolic groups, delocalization of free radicals occurred from the phenolic structure [31]. It has been proved to have potential therapeutic effects in many diseases where the oxidative stress has been implicated. Antioxidant activity was evaluated for indole moiety in gallic hydrazones [32]. About 33 gallic acid derivatives were synthesized and investigated for their potential antibacterial and antifungal activities [33]. Azo gallic acid complexes were reported for their promising antimicrobial activity [34]. Gallic acid-based indanone derivatives were reported to have anticancer activity [35]. Some compounds such as S-(3,4-methylenedioxyphenyl)-3,4,5-trihydroxy-thiobenzoate (GD-3) and 3,4-methylenedioxyphenyl 3,4,5-trihydroxybenzoate (GD-1) derivatives of gallic acid showed significant cytotoxicity in cancer cell lines [36].

Our research work has some significances and limitations. This is the first research activity on* Ficus racemosa* which focused on the correlation of the presence of polyphenolic compounds with its pharmacological activities. Moreover, our findings supported the traditional use of this plant which is worth further large scale investigation. However, the potential limitations of this research work are as follows: first, we used brine shrimp to investigate the cytotoxic effect rather than using animal models or cancer cell line. Secondly, we used only two doses and ten bacterial strains for antibacterial investigation which is not enough. As the plant showed a dose dependent antibacterial effect, increasing dose as well as using a good number of bacterial strains may provide a better picture.

## 5. Conclusion

The methanol extract of the leaf and fruit part of* Ficus racemosa* exhibited significant antioxidant activity. The extracts also showed significant antibacterial and cytotoxic activities. The observed pharmacological activity may be due to the presence of significant concentration of gallic acid in the plant extracts. In addition, other phenolic constituents present in the plant might have assisted towards these pharmacological activities.

## Figures and Tables

**Table 1:** Total phenolic, flavonoid, and tannin content of the extracts.

Extract	Total phenolic content (mg GAE/g of dry extract)	Total flavonoid content (mg QE/g of dry extract)	Total tannin content (mg GAE/g of dry extract)
LCME	20.2	22.81	19.72
FCME	26.2	10.63	21.39

**Table 2:** In vitro antibacterial activity of methanol crude leaf extract of *Ficus racemosa*.

Bacterial strains	Type of bacterial strains	Diameter of zone of inhibition (mm)		
		Standard (Kanamycin 30 *μ*g)	Extract 250 *μ*g	Extract 500 *μ*g
*Vibrio cholerae*	Gram (−)	26.7	—	—
*Shigella dysenteriae*	Gram (−)	21.14	—	—
*Escherichia coli*	Gram (−)	23.62	5.10	9.61
*Shigella sonnei*	Gram (−)	26.34	—	—
*Shigella flexneri*	Gram (−)	27.16	7.13	10.08
*Shigella boydii*	Gram (−)	22.80	6.13	8.45
*Enterococcus faecalis*	Gram (+)	31.73	—	—
*Staphylococcus aureus*	Gram (+)	29.53	—	—
*Staphylococcus epidermidis*	Gram (+)	27.41	2.15	4.23
*Streptococcus pyogenes*	Gram (+)	25.32	—	—

**Table 3:** In vitro antibacterial activity of methanol crude fruit extract of *Ficus racemosa*.

Bacterial strains	Type of bacterial strains	Diameter of zone of inhibition (mm)		
		Standard (Kanamycin 30 *μ*g)	Extract 250 *μ*g	Extract 500 *μ*g
*Vibrio cholerae*	Gram (−)	26.7	—	—
*Shigella dysenteriae*	Gram (−)	21.14	—	—
*Escherichia coli*	Gram (−)	23.62	3.15	7.41
*Shigella sonnei*	Gram (−)	26.34	—	—
*Shigella flexneri*	Gram (−)	27.16	6.38	8.23
*Shigella boydii*	Gram (−)	22.80	6.57	8.68
*Enterococcus faecalis*	Gram (+)	31.73	—	—
*Staphylococcus aureus*	Gram (+)	29.53	—	—
*Staphylococcus epidermidis*	Gram (+)	27.41	3.13	5.27
*Streptococcus pyogenes*	Gram (+)	25.32	—	—

**Table 4:** Contents of polyphenolic compounds in the methanol extract of *Ficus racemosa* leaves (*n* = 5).

Polyphenolic compound	Methanol extract of *Ficus racemosa* leaf	
	Content (mg/100 g of dry extract)	% RSD
AR	23.91	0.25
GA	37.82	0.35
CH	16.91	0.17
VA	14.07	0.13
CA	11.82	0.11
ECA	20.75	0.19
VL	5.02	0.05
FA	5.44	0.06

RSD: relative standard deviation.

**Table 5:** Contents of polyphenolic compounds in the methanol extract of *Ficus racemosa* fruit (*n* = 5).

Polyphenolic compound	Methanol extract of *Ficus racemosa* fruit	
	Content (mg/100 g of dry extract)	% RSD
GA	50.11	0.42
CH	25.34	0.19
VA	16.38	0.14
CA	12.06	0.11
SA	2.97	0.04
ECA	22.14	0.16
VL	7.61	0.06
PCA	2.93	0.03
FA	6.05	0.05
RH	4.74	0.04

RSD: relative standard deviation.
